# Association between Traditional Risk Factors and Coronary Artery Ectasia: A Study on 10057 Angiographic Procedures among Iranian Population

**Published:** 2014-01-12

**Authors:** Ali Reza Amirzadegan, Gholamreza Davoodi, Abbas Soleimani, Masoumeh Lotfi Tokaldany, Elham Hakki Kazazi, Hoda Shabpiray, Mani Khorsand Askari

**Affiliations:** Tehran Heart Center, Tehran University of Medical Sciences, Tehran, Iran.

**Keywords:** *Coronary angiography* • *Coronary vessel anomalies* • *Prevalence* • *Risk factors*

## Abstract

***Background: ***Whether coronary artery ectasia (CAE) is a unique clinical finding or results from other clinical entities is still unknown. We aimed to determine the CAE prevalence, investigate the relationship between CAE and patients’ demographic and clinical characteristics, and assess the prognosis at follow-up in a sample of Iranian population.

***Methods:*** Totally, 10057 patients who underwent coronary angiography were divided into three categories: normal coronary arteries without co-existing coronary artery disease; CAE without co-existing coronary artery narrowing < 50%; and coronary artery stenosis with > 50% luminal narrowing (CAS).

***Results:*** The prevalence of CAE was 1.5%. Compared to the normal individuals, the CAE patients were older, were more frequently male, and had higher rates of myocardial infarction (MI). The CAE patients had a lower frequency of diabetes and MI than the CAS group. The CAE patients were largely focused between 40 to 60 years of age. The right coronary and left anterior descending arteries were the most involved arteries, and ectasia was located more frequently in the proximal part of these arteries. Patients with ectasia in the three main vessels had higher rates of MI. After a mean follow-up of 54.23 ± 18.41 months, chest pain and dyspnea on exertion remained the main complaint in more than 97% of the patients, leading to hospital admission in more than 14%.

***Conclusion: ***There was no relationship between the presence of ectasia and conventional risk factors. According to our study, pure CAE may be deemed a benign feature of atherosclerosis; however, it can lead to frequent hospital admissions because of the persistence of cardiovascular symptoms.

## Introduction

Coronary artery ectasia (CAE), also known as dilated coronopathy, is a relatively uncommon angiographic finding.^1^^-^^3^ This condition is diagnosed when the diameter of a dilated segment of an artery is 1.5 times greater than the diameter of the adjacent normal segments of the artery.^2^ According to the findings of autopsies and those during coronary angiography or multi-detector computed tomography, there is a wide variation in the incidence of CAE (between 0.3% and 12%),^1^^, ^^2^^, ^^4^^, ^^5^ depending on the methodology and the population selected. 

There are various methods for defining CAE according to the severity and extent of this condition. According to the study method, different descriptions of CAE have been introduced. Hartnell et al.^2^ defined CAE as an arterial segment with a diameter at least 1.5 times the diameter of the adjacent normal coronary artery. Markis et al.^6^ introduced the following classification of CAE based on the extent of coronary involvement: type I, diffuse ectasia of two or three vessels; type II, diffuse disease in one vessel and localized disease in another vessel; type III, diffuse ectasia of one vessel only; and type IV, localized or segmental ectasia. This classification is also modified in some studies as it is not always possible to group all the patients as per the Markis^7^ classification. 

Regardless of the severity and extent of CAE, the etiology, prognosis, morbidity, and mortality related to this abnormality are still a matter of debate and whether CAE is a unique clinical finding or a state resulting from other clinical entities is still unknown. However, several investigations have suggested that congenital, inflammatory, and connective tissue disorders are possible etiologies and that the atherosclerotic process is the main cause in the majority of the cases.^2^^, ^^8^^, ^^9^ In addition, the prognosis differs significantly between studies,^10^^, ^^6^ with the annual mortality rate having been reported between 2% to 15%.

The aim of this study was, firstly, to determine the prevalence of CAE amongst patients who underwent coronary angiography for an investigation of coronary artery stenosis. We also sought to evaluate the possible relationship between the existence of CAE without significant coronary artery stenosis and the patients’ demographic and clinical characteristics by comparing the affected individuals with a group of subjects with normal coronary arteries and a group of patients with coronary artery stenosis > 50 narrowing in at least one vessel. Additionally, we followed up the CAE patients without significant coronary artery stenosis for their prognosis. 

## Methods

We retrospectively assessed data from 12514 patients who underwent coronary angiography, for different reasons, by three experienced staff cardiologists between January 2005 and January 2011 in Tehran Heart Center, Tehran, Iran. The investigation was approved by the institutional Review Board, overseeing the participation of human subjects in research at Tehran University of Medical Sciences. This manuscript was drafted in accordance with the European Association of Science Editors’ guidelines for authors of scientific articles.^11^

The indications for coronary angiography were a history of angina, previous or an acute myocardial infarction (MI), and positive noninvasive diagnostic tests. Patients with congenital or valvular heart disease and cardiomyopathy were excluded, leaving 10057 patients for further evaluation. Therefore, these patients underwent coronary angiography exclusively for an investigation of coronary stenosis. 

A vessel was considered ectatic if its luminal diameter was > 1.5 times that of the adjacent normal segment.^4^ Coronary artery stenosis (CAS) was defined as the existence of > 50% stenosis in the coronary lumen, while < 50% stenosis was considered non-significant.

According to the results of angiography, the patients were categorized in three groups: 1) normal coronary arteries without co-existing coronary artery narrowing; 2) CAE without any co-existing coronary artery narrowing > 50%; and 3) coronary artery stenosis (> 50% luminal narrowing). 

The distribution of ectasia in coronary vessels was defined as proximal, mid, and distal portions in the left anterior descending artery (LAD), left circumflex artery (LCX), and right coronary artery (RCA). The presence of ectasia in the major branches and also in the left main coronary artery (LM) was evaluated. The ejection fraction for all the patients was also determined by ventriculography.

Demographic, clinical, and procedural information was obtained from the Angiography Databank of our institution. Data on noninvasive tests such as surface electrocardiography, exercise tolerance test, and myocardial perfusion imaging were also extracted from the Databank only for the CAE group. The definitions of the variables in our Databank have been previously reported.^12^ In summary, the validation of acute myocardial infarction (MI) events was based on information on medical history, symptoms, electrocardiogram, and cardiac enzymes. Coronary artery disease (CAD) risk factor profile, comprised of history of cigarette smoking (patient regularly smokes cigarette one or more times per day or has quit smoking during the last 24 months), hyperlipidemia [total cholesterol ≥ 200 mg/dl, high density lipoprotein (HDL) ≤ 30 mg/dl and triglycerides ≥ 150 mg/dl], family history of CAD (first-degree relatives before the age of 55 in men and 65 years in women), hypertension (systolic blood pressure ≥ 140 and/or diastolic ≥ 90 mmHg and/or on anti-hypertensive treatment), diabetes mellitus [symptoms of diabetes and plasma glucose concentration ≥ 200 mg/dl (11.1 mmol/l)], and fasting blood sugar (FBS) ≥ 126 mg/dl (7.0 mmol/l) or 2-hp ≥ 200 mg/dl (11.1 mmol/l). The body mass index (BMI) was calculated (via formula: weight/height^2^, kg/m^2^), and all the patients with BMI ≥ 25 kg/m^2^ were defined as obese. 

The patients in the CAE group were followed up and were asked to be visited in the outpatient clinics. Follow-up data included symptoms, history of hospitalization, and cardiovascular and non-cardiovascular death.

For the statistical analysis, the statistical software SPSS version 20.0 for Windows (SPSS Inc., Chicago, IL) was used. The continuous variables are presented as mean ± standard deviation (SD), while the categorical variables are summarized by percentages. The continuous variables were compared using the Student *t*-test or the Mann-Whitney U, and the categorical variables were compared using the chi-squared test. As we used registered data in the databank, some of the patients had missing data on risk factors at a low rate between 0.4% and 3%. Data for height and weight were not available for 7.5% of the patients. All the p values were two-tailed, with statistical significance defined by a p value ≤ 0.05.

## Results

Amongst the 10057 patients in our study population, 229 patients had CAE, showing a prevalence of 2.3%. There were 6705 (66.7%) male and 3352 (33.3%) female cases. The prevalence of total CAE (CAE with or without luminal narrowing > 50%) amongst the male patients was 2.7% (183/6705) and that of the female patients was 1.4% (46/3352), showing a twofold higher probability of CAE in the men (p value < 0.001). In the CAE patients, 78/229 (34.1%) cases had co-existing luminal narrowing > 50% and were excluded from further analysis because the main focus of the study was on pure ectasia patients, who may be different from those having CAE in addition to CAS. The remaining 151 CAE patients did not show concomitant luminal narrowing > 50%; therefore, the prevalence of CAE without luminal narrowing > 50% was 1.5% in the whole study population. 

We categorized our patients in three groups (described in the method section). There were 10057 angiograms: 8164 (81.2%) patients had coronary artery stenosis > 50%; 78 of these patients who had CAE were excluded. Therefore, a total of 8086 patients were included as the CAS group (without CAE). The normal group consisted of 1742 patients and the CAE group included 151 patients. These three groups were compared regarding their demographic and clinical characteristics. 

The demographic and clinical findings of both groups are depicted in Table 1. The patients with CAE were significantly older than those with normal coronary arteries. The CAE group was more likely to be male and smoker than the normal group. When we divided the patients according to their gender, there was no significant difference in the proportion of smokers between the men with a normal coronary artery and the men with CAE, and similar result was observed in the women. The prevalence of diabetes mellitus, hypertension, hyperlipidemia, and family history of coronary artery disease was similar in both CAE and normal groups. The mean of the BMI and the rate of obesity did not differ between the groups (84.5% in CAE and 81.1% in normal groups, respectively). 

Regarding the presenting symptoms in each group (Table 1), the CAE patients were less likely to present with chest pain; however, this group had a higher rate of a history of MI. The mean ejection fraction was lower in the CAE patients. 

A total of 777 (44.5%) normal individuals and 55 (36.4%) CAE patients underwent the exercise tolerance test (ETT) and 697 (40.0%) normal and 56 (37.1%) CAE cases underwent myocardial perfusion scan prior to angiography. The result of the ETT was positive in 80.7% of the normal coronary and 75% of the CAE patients (p value = 0.301). Myocardial perfusion scan was positive in 79.7% and 82.1% for the normal and CAE patients, respectively (p value = 0.670).

The demographic and clinical findings of both groups are compared in Table 1. The patients with CAE were younger than those with CAD, while gender distribution was similar in both. The CAS group had a higher frequency of diabetes mellitus. The prevalence of hyperlipidemia, family history of CAS, and smoking was similar in both groups. More patients in the CAE group were overweight; 84.5% in the CAE and 71.2% in the CAS groups had BMI > 25, respectively. The CAS group had a lower ejection fraction, and the rate of MI was 3 times higher than that of the CAE group. From 8086 CAS patients, 3.5% had a history of percutaneous coronary intervention and 3% had a history of coronary artery bypass graft surgery.

A total of 1817 (22.5%) CAS patients underwent the ETT and 1478 (18.3%) underwent myocardial perfusion scan prior to angiography. A positive ETT was more prevalent in the CAS group than in the CAE patients (89.1% vs. 75%, respectively; p value = 0.001). Also, a positive myocardial perfusion scan was significantly higher in the CAS group than in the CAE group (90.9% vs. 82.1%; p value = 0.027, respectively).

Of 151 individuals (mean age: 56.97 ± 10.86 years, range: 33 to 75 years) with ectasia without coronary narrowing > 50%, 113 (74.8%) were male and 38 (25.2%) were female. Further analysis on the CAE group showed that the age of the patients was more likely to be between 40 and 60 years (56.3%, 85/151). The number of the patients aged > 60 years was 53/151 (35.6%) and that of the patients aged < 40 years was 13/11 (8.7%). Amongst the CAE patients, 74.5% presented with chest pain or exertional dyspnea (Table 1).

The RCA and LAD were the most common involved vessels. Ninety-three (61.6%) patients had involvement of the RCA, 92 (60.8%) cases had ectasia in the LAD, and 55 (36.4%) showed ectasia of the LCX. Eleven (7%) patients had concomitant involvement of three vessels (LAD, RCA, and LCX, or their branches). Amongst these 11 patients, 2 also had the involvement of the left main coronary artery. Ectasia of two vessels was found in 67 (44.4%) patients, 11 of whom also had the involvement of the left main coronary artery. The presence of ectasia in only one major vessel was observed in 73 (48.3%) patients: 31 (20.5%) cases in the RCA; 27 (17.9%) in the LAD; and 15 (9.9%) in the LCX. Table 2 presents the frequency of CAE in the entire coronary arterial system. As regards the proximal, mid, and distal portions of the affected arteries, the most common affected portion was proximal RCA (57%) and proximal LAD (52.3%). A history of MI was found in 27.3% of the patients with three involved vessels, 11.9% of those with two involved vessels, and 13.7% of the ones with one involved vessel, without there being any significant difference.

**Table 1:** Baseline characteristics of the study groups^*^

				P value	P value
	Normal group n=1742	CAS group n=8057	Ectasia group n=151	CAE vs. Normal	CAE vs. CAS
Age (y)	54.01±9.75	59.21±9.96	56.97±10.86	< 0.001	0.030
Male sex	771 (44.3)	57517 (71.1)	113 (74.8)	< 0.001	0.318
BMI (Kg/m^2^)	29.22±4.96	27.51±4.30	29.28±4.55	0.875	< 0.001
Hyperlipidemia	1080 (63.0)	5399 (67.5)	102 (68.9)	0.150	0.706
Hypertension	807 (46.6)	4247 (52.6)	67 (44.7)	0.654	0.052
Diabetes mellitus	315 (18.2)	2545 (31.5)	29 (19.6)	0.668	0.002
Cigarette smoking	357 (20.5)	3383 (41.8)	54 (35.8)	< 0.001	0.134
Family history of CAD	363 (21.5)	1787 (22.62)	29 (19.5)	0.555	0.371
Ejection fraction (%)	60.13±4.72	49.78±11.33	58.89±5.31	0.005	< 0.001
Presentation					
Chest pain	1368 (78.8)	5312 (66.2)	105 (69.5)	0.008	0.392
Dyspnea on exertion	120 (6.9)	538 (6.7)	7 (4.6)	0.287	0.312
History of myocardial infarction	93 (5.3)	3471(42.9)	21 (13.9)	< 0.001	< 0.001

CAS, Coronary artery stenosis; CAE, Coronary artery ectasia; BMI, Body mass index

* Data are presented as mean±SD or n (%)

**Table 2:** Distribution of coronary ectasia in different vessels of 151 patients

Affected vessel	Number	Percentage
RCA		
Proximal RCA	86	57%
Mid RCA	58	38.4%
Distal RCA	25	16.6%
LAD		
Proximal LAD	79	52.3%
Mid LAD	35	23.2%
Distal LAD	4	2.6%
Diagonal branch	4	2.6%
LCX		
Proximal LCX	43	28.5%
Obtuse marginal branch	16	10.6%
Left Main	15	9.9%

RCA, Right coronary artery; LAD, Left anterior descending; LCX, Left circumflex

The patients with CAE were followed up regarding their symptoms, hospital admissions, major cardiovascular events, and cardiovascular and non-cardiovascular death. Out of the 151 cases, 131 patients were successfully followed up at a rate of 86.8%. The mean follow-up time was 54.23 ± 18.41 months, ranging between 20 and 85 months. During the follow-up period, 128 (97.7%) patients still had chest pain (n = 115, 76.2%) or suffered from dyspnea on exertion (n = 13, 8.6%). Three (2%) cases experienced syncope. Amongst the symptomatic patients, 19 (14.5% of a total of 131 patients) had hospital readmission. Two patients were hospitalized twice. None of the patients died due to cardiovascular events during the course of follow-up; nevertheless, 2 patients died: one due to a car accident and the other one expired in the hospital due to sudden death a day after angiography. 

## Discussion

According to the findings of this study, the prevalence of CAE amongst a large population of patients with suspicion of CAD was 2.3% and this figure for ectasia without narrowing > 50% in coronary arteries was 1.5%. Compared to those with a normal coronary artery, our CAE patients were older, more frequently male, and more likely to have a history of MI. Compared to the patients with CAS, the CAE patients had a lower frequency of diabetes mellitus and MI but with a higher probability of obesity. Additionally, 91.9% of the CAE patients were over 40 years old and they were largely focused between 40 to 60 years of age. The RCA and LAD were the most involved arteries and ectasia was located more in the proximal part of these arteries. A mean follow-up of 4.5 years after angiography showed that chest pain and dyspnea on exertion remained the main complaint in more than 97% of the patients, leading to hospital admission for more than 14%. No cardiac-related death was reported.

The prevalence of CAE varies depending on the series. In our study, after the exclusion of patients with valvular, congenital heart disease, and cardiomyopathy, the rate of CAE in the patients who underwent coronary angiography exclusively because they were suspected of having coronary stenosis was 2.3%. With a similar setting, Pinar Bermudez et al.^13^ found 3.39% CAE prevalence in Spanish patients. Lam et al.^3^ found a prevalence of 1.2% amongst all the patients who underwent coronary angiography in Singapore, while in the same setting, Giannoglou et al.^14^ reported a prevalence of 2.7% in a sample of Greek population. The rate for a sample of Indian population was much higher (12%) according to the findings of the Sharma et al.^4^ study. Meanwhile a few studies have explored the rate of CAE without luminal narrowing > 50%. In a study by Giannoglou et al.,^14^ the rate of CAE in 2150 patients without luminal narrowing > 50% was 1.7%. The Nyamu et al.^7^ study excluded patients with significant coronary stenosis and reported a prevalence of 1.9% for isolated CAE in patients without coronary artery narrowing > 50%. In the present study, the frequency of CAE without narrowing > 50% was 1.4% in a sample of Iranian population who underwent coronary angiography on suspicion of coronary stenosis. 

CAE was remarkably predominant in our male compared to female patients (2.7% for men vs. 1.4% for women). This is in line with previous findings which highlighted the male preponderance for CAE, with a male-to-female ratio of 2:1.^14^ The reported ratio in Singapore was higher 3:1, although the incidence of CAE amongst men and women was lower than that of our study.^3^ This may be because the authors did not exclude any patients from the registry, whereas we excluded patients with valvular, congenital, and cardiomyopathy disease. Nevertheless, a study in Spain also found the male gender as an independent predictor of CAE.^13^

The age of the CAE patients without coronary narrowing > 50% in our study was significantly lower than that of the patients with coronary stenosis > 50%. This finding confirms the results of some previous studies.^13^^, ^^14^

Many other clinical entities other than atherosclerosis can cause dilation of the coronary arteries such as syphilis, mycotic or bacterial infection, Kawasaki disease, trauma, congenital heart disease, inflammatory disorders, connective tissue disorders like Marfan's syndrome, scleroderma, systemic lupus, Ehlers-Danlos' syndrome, periarteritis nodosa, Behcet’s disease, and congenital defects.^3^^,^^15^^-^^18^ None of the above etiologies were identified in our study patients to be available in our Angiography Databank. Differentiation between the congenital and atherosclerotic forms of CAE is difficult, and diagnosis of CAE at a very young age in the absence of other etiologies may be suggestive of congenital form. In the present study, the minimum age of the patients with pure CAE was 33 years and atherosclerotic pathology could not be refused. 

Thus far, no specific risk factor has been identified and most studies have not found any correlation between CAE and traditional cardiovascular risk factors.^12^^, ^^18^ Be that as it may, one study suggested that hypercholesterolemia could be a predisposing factor.^19^ Two studies determined a negative correlation between diabetes mellitus and CAE.^14, 20^ In our study, there was no apparent correlation between CAE and traditional cardiovascular risk factors. Although compared to the normal coronary patients, the CAE group had a higher rate of cigarette smoking, this is due to the fact that men are more likely to be smokers than women and the proportion of men in the CAE group was much higher than that in the normal coronary group. 

Similar to other pervious reports, the RCA was the most commonly affected vessel in our study^3^^, ^^4^ and the involvement of the LAD was not much less than that of the RCA (61.6% for the RCA and 60.8% for the LAD). This finding does not chime in with that of the Nyamu et al.^7^ study, where the LAD was the most common affected vessel. The authors in question reported that ectasia in the LAD showed almost a discrete form of ectasia, while the RCA had predominantly a diffuse feature, as was described by Demopoulos VP et al.^21^

A considerable proportion of our CAE patients [26 (17.2%)] had a history of MI or presented with acute MI, while this figure was only 5.4% in the patients with a normal coronary artery, as was expected.^22^ Nyamu et al.^7^ and Demopoulos et al.^21^ reported a higher frequency of MI history in patients with isolated CAD than what we observed in our study. Nonetheless, these observations have convinced investigators that CAE may not be a benign condition and the patients are at risk for MI and sudden cardiac death due to slow flow, coronary vasospasm, dissection, and/or intracoronary thrombosis.^23^

Our results of over 4 years' follow-up on 131 patients with isolated CAE showed that although the majority of our patients continued to struggle with their chest pain and dyspnea on exertion, leading to hospitalization for 14.5%, none of the available patients had MI or cardiac death or required any intervention. Similar findings were reported by Nyamu et al.^7^ and Demopoulos et al.^21^

## Conclusion

We conclude that there is no relationship between the presence of ectasia and conventional risk factors. Although isolated CAE may be considered a benign feature of atherosclerosis, it can lead to frequent hospital admissions and more days off work because of the persistence of cardiovascular symptoms, which may be prevented by better and more specific considerations. 

## Notes

***This paper should be cited as:*** Amirzadegan AR, Davoodi G, Soleimani A, Lotfi Tokaldany M, Hakki Kazazi E, Shabpiray H, Khorsand Askari M. Association between Traditional Risk Factors and Coronary Artery Ectasia: A Study on 10057 Angiographic Procedures among Iranian Population. J Teh Univ Heart Ctr 2014;9(1):27-32.
